# Fair Trade Metaphor as a Control Privacy Method for Pervasive Environments: Concepts and Evaluation

**DOI:** 10.3390/s150614207

**Published:** 2015-06-16

**Authors:** Abraham Esquivel, Pablo Haya, Xavier Alamán

**Affiliations:** 1 Instituto Tecnológico Superior Zacatecas Norte, Río Grande (Zac) 98400, Mexico; E-Mail: abraham.esquivel@itszn.edu.mx; 2 Instituto de Ingeniería del Conocimiento, Universidad Autónoma de Madrid, Madrid 28049, Spain; E-Mail: pablo.haya@iic.uam.es; 3 Escuela Politécnica Superior, Universidad Autónoma de Madrid, C. Francisco Tomás y Valiente. 11, Madrid 28049, Spain

**Keywords:** fair trade metaphor, privacy management, ambient intelligence

## Abstract

This paper presents a proof of concept from which the metaphor of “fair trade” is validated as an alternative to manage the private information of users. Our privacy solution deals with user's privacy as a tradable good for obtaining environmental services. Thus, users gain access to more valuable services as they share more personal information. This strategy, combined with optimistic access control and transaction registry mechanisms, enhances users' confidence in the system while encouraging them to share their information, with the consequent benefit for the community. The study results are promising considering the user responses regarding the usefulness, ease of use, information classification and perception of control with the mechanisms proposed by the metaphor.

## 1. Introduction

Ambient intelligence is a promising research area that opens attractive perspectives for improving human-computer interaction. Since AmI systems require knowing user's private information, privacy issues are especially relevant and the subject of an active discussion [1–3]. Several technological methods have been used by organizations to address privacy [4,5] as result of the mix between computer security and cryptography fields [6]. In this sense, a major step in leveraging AmI comes from overcoming their legal [7], ethical and psychological issues [8].

Privacy is a dynamic phenomenon [9,10]; its configuration has as many variations as variations have contexts, *i.e.*, a single change in context can trigger a change in privacy preferences. As a consequence, privacy management should be a continuous negotiation in which the definition of the public and private boundaries will depend directly on the user's context. Therefore, privacy management is a constant process of limit regulation. Those limits to accessibility of personal information determine the “sincerity”, or “openness”, or “distrust”, or “closeness” characterizing the user and his/her current context.

However, how can the user establish those limits? A first approximation can be to manually configure privacy, assigning the desired level to each source of information. The key problem of this solution relays on the nature of privacy and pervasive environments: the degree of privacy desired for a source of information depends not only on the user and the source, but also on the context. In other words, for every source and every person, there can be as many different privacy configurations as different contexts in which they can be involved. Configuring *a priori* each possible arising situation for every source of information can be an overwhelming task that justifies the use of automatic management solutions.

The success of such automatic management solutions depends directly on the trust the user place in them. This trust depends on the following requirements: (1) the model must be simple enough to be understood by a non-technical user; and (2) the user must be able to modify the automatic configuration at any moment. In developing our framework, we focused particularly on the importance of usability. Especially in this case, confidence and trust are synonyms of usability.

Following this premises, we present a privacy solution dealing with user's privacy as a tradable good for obtaining environmental services. Thus, users gain access to more valuable services as they share more personal information. This strategy enhances users' confidence in the system, while encouraging them to share their information.

This paper presents a proof of concept to validate our framework, which is called the “fair trade” metaphor. Our framework is a managing privacy proposal for environments where violation is not intentional, but a product of interaction between users and those with an active environment.

## 2. State-of-the-Art

There are a variety of privacy definitions in the literature; as a controversial word, it is used under different scenarios and contexts, always present when it is required to justify the confidentiality of information.

– Cardoso [11] defines privacy as “control over information disclosure”.
– Culnan [12] makes another simple and useful proposal, “privacy as the right to control information about oneself”.
– To Leysia Palen and Paul Dourish [13], privacy is a dynamic process, subject to negotiation and ongoing management, with a limit that distinguishes what is private from public, according to the circumstances.

Some aspects concerning privacy, such as fundamentals for its treatment [2], controversies [14], proof of concepts [15], including challenges when it is necessary to design a tool for preserving privacy [16], have been widely discussed.

Price in [17], presents a good alternative to classify the background in the pervasive computing research area. His proposal is to create two groups: the first one is focused on policy matching techniques, and the second one tries to hide or disguise a user's location or identity (noise-anonymizing, hashing, cloaking, blurring, lying).

Geopriv is a standard for the transmission of location information over the Internet [18]. The work in the IETF Geopriv working group particularly focused on the privacy and security issues, both from a technology perceptive and a policy perspective, of sharing location information over the Internet. Actually, it supports a rich set of XML rules that permit users to grant or deny access to their location information. Additionally, Geopriv can also specify the level of detail with which one can display location information (noise in the form of blurring).

Bagüés in [19] present the CONNECT Platform. Connect defines a common software platform for mediating between users, their privacy needs, privacy management and the context-aware mobile services. Connect will support privacy policy matching, obfuscation and identifier abstraction, which hides the target's true identity.

The PRIME Project (Privacy and Identity Management for Europe) [20] has designed and implemented an identity management system. The architecture handles access control, advanced anonymous credential systems and automated reasoning. PRIME allows users to share credentials and personal information with different transaction partners, in the presence of a variable level of trust.

The RAVE project (Ravenscroft Audio Video Environment) is a media space created to communicate with geographically-dispersed people. In RAVE, cameras, monitors, microphones and speakers are placed in every office, to provide everyone with their own personal RAVE node. This allows one to communicate and work with others and to be aware of what is going on in the building without leaving one's office [2,21]. The privacy protection is done through the control principle (empowering people to stipulate what information they project and who can get a hold of it) and feedback (informing people when and what information about them is being made available). As a main RAVE benefit, users can control who may connect to them and what kind of connections each person is allowed to make. If they decline to do so, automatic defaults are set to reject connections.

The privacy awareness system (pawS) introduces a privacy awareness system targeted at ubiquitous computing environments that allow data collectors to both announce and implement data usage policies, as well as providing data subjects with a technical means to keep track of their personal information as it is stored, used and possibly removed from the system [22]. pawS privacy proxies check these policies against user's predefined privacy preferences. Services can collect information, and users can utilize these services if the policy agrees.

PerGym provides personalized services on the basis of sensitive context data [23,24]. In the PerGym scenario, users of the gym carry a mobile device (e.g., their smartphone, PDA or a smart watch provided by the gym) that collects context data from environmental and body-worn sensors to continuously monitor data, such as user's position (acquired through a user-side indoor positioning system), used equipment (through RFID), emotional status, physical activity and physiological parameters. A part of these data is communicated by users to the gym service provider included in requests to obtain personalized services. The proposed architecture supports the acquisition of context data from different sources, the reasoning with these data based on distributed policies and the reconciliation of possibly conflicting information. In order to protect users' privacy, request are sent through an encrypted channel to a context-aware privacy module (CPM) in charge of enforcing users' privacy policies.

Daidalos is a European research project in the area of 3G and beyond, which aims to combine heterogeneous networks in a transparent and seamless way and to develop on top of this a pervasive environment for applications and end-users. The privacy protection mechanisms applied in the Daidalos context management system fall mainly into two categories [25,26]. On the one hand, an access control mechanism has been established that requires authentication and authorization verification of the party requesting context information. Once the necessary access rights are in place, the system delivers the requested context data. On the other hand, pseudonymization of context identifiers is applied, creating virtual identities.

Privacy Violation Avoider (PriVA) is a privacy-aware model [27], aimed at avoiding privacy violation for resource sharing. PriVA works by detection of privacy violation based on policy and is flexible, as it works with default policies (users do not need to assign anything) and also user-defined policies (some users may want to assign policies by themselves). Furthermore, the model has a list of custom policies available to the user, providing flexibility in their policies.

MIX networks and MIX nodes [28,29] provide a strategy to protect the information of possible malicious observers, making it impossible to trace the information from the service provider to the client. The messages enter a network of MIX-servers in an appropriate order to get to the final destination. At each MIX-server visited, a message is recoded appropriately, eliminating the possibility of tracing between the incoming message and outgoing message.

The hierarchical identity-based encryption [30,31], implemented in the context of the Aura pervasive computing environment, proposes an access control mechanism in which depending on a set of permissions, the resolution of the information (granularity) that travels from sender to receiver is obtained. The resolution's information and privacy levels are specified from rules defined by the owner of the information.

Privacy-protecting middleware [11] proposes an architecture that provides mechanisms to control the disclosure of personal information and to enable users to reveal only the information strictly necessary to perform service access. This can reduce the effects of usage violations of personal data executed by malicious third parties. The service-oriented infrastructure layer contains modules to publish, discover, compose and access services. The middleware sits in between two layers, mediating requests for contextual data and communication. It enforces access control whenever data are accessed and transmitted and adapts contextual information to protect the user's personal data.

SecSpace [32] is a framework for exploring usable privacy and security mechanisms for mixed reality collaborative environments. It manage privacy in mixed reality collaborative spaces, through five strategies: (1) privacy mechanisms that are appropriate to the physical and virtual worlds; (2) visually representing the current policies in both worlds; (3) building on social norms when negotiating privacy mechanisms between the worlds; (4) enforcing privacy mechanisms based on context; and (5) providing simple authentication and permission controls. SecSpace's ontology is used for reasoning across objects and events in both spaces, for example to infer activity. Rules are evaluated that can generate commands to specific entities or classes of entities. SecSpace uses this feature to define privacy rules once for both physical and virtual spaces, to link shared resources that have physical and virtual manifestations and to respond to contextual events that can occur in physical, virtual or both spaces simultaneously.

WIPR [33] (Weizmann-IAIK (Institute for Applied Information Processing and Communications) Public Key for RFID, a low-resource, public-key encryption scheme)-enabled tags show a practical design for a secure RFID supply-chain system that uses public key cryptography. They are fully compatible with the existing ecosystem of non-secure tags, readers and terminals. Their use of public-key cryptography reduces trust issues between the supply-chain owner and tag manufacturer, ensures that reverse-engineered tags do not compromise the whole system's security and protects user privacy. They conclude that the public-key approach is a viable design alternative for supply-chain RFID tags based on the Electronic Product Code (EPC) standard.

Percontrol [34] is a system that manages and keeps track of user attendance in an automatic way; in other words, the system detects the entrance and exit of users within an environment, be it academic or entrepreneurial. Percontrol also bolsters a user discovery and localization service, within the local environment, that is based on Bluetooth, WiFi and RFID. The system identifies users through their mobile devices and controls the environment (temperature, for example) based on their previously-defined profiles. Future work needs to define a model and implement a mechanism for privacy control, for both users, their devices, as well as for the pervasive environment itself.

Each of these methods is designed to balance the need between privacy protection and the quality of service provided to the user. It is important to remember that pervasive environments require privacy solutions that are especially flexible. Too much detail or too many restrictions in every situation configuration can end up oppressing the end-user. Our proposal is to motivate users to configure their privacy in order to obtain more and better services from the environment, propitiating the creation of affinity groups from where to obtain feedback about what, when and to whom information is delivered. In our approach, context information is filtered and in charge of adjusting the granularity with which information is delivered. Due to the “fair trade” policy, filtering is a matter of two factors: the access granted by the owner and the privacy settings of the inquirer.

## 3. Proposal

### 3.1. Privacy Framework

Privacy can be seen as a matter of information, entities and their relation. Every privacy solution has to deal with these factors and their characteristic problems. In this section, we will analyze the elements and factors involved in our privacy world.

Information is probably the most important element of privacy. In fact, privacy can be summarized as protecting information loosely enough to permit interaction, but sufficiently tight to preserve confidentiality. When talking about pervasive environments, in which all of the information is digital, and thus storable, it is important to think about the use of some information not only in the present, but also in the future.

From the privacy management point of view, several elements can be distinguished:

– User: an entity with administrative rights; it can be a single person or an organization.
– Agent: a software module that acts on behalf of other persons or organizations.
– Space: a physical or virtual area.
– Data source: a resource providing information about the environment's entities; data can vary from a single value, such as temperature, to a multimedia streaming.
– Data source owner: the user with administrative rights on the data source.
– Inquirer: the agent or user inquiring about data source.
– Receiver: the agent or user receiving the answer from the inquirer; in some instances, it is possible that the receiver and the inquirer are not the same entity, as for example when the inquirer's answer is broadcast to a group of receivers.

**Figure 1:**
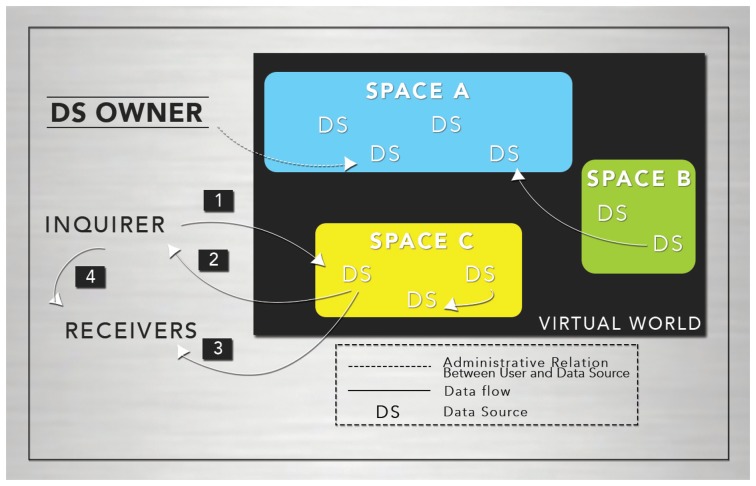
Graphical representation of the privacy framework.

In Figure 1, we provide a high-level illustration of our design framework. As shown, we consider multiple spaces organized hierarchically. Each of them represents a physical or conceptual space serving as container of data sources [35].

Additionally, every data source is represented in the virtual world, where it can also store information (see Figure 1). This is the case of the RFID card reader installed in our laboratory. This card reader generates location information and stores it in the virtual world representation.

We assume that every data source providing information of a user is controlled by him/her. Additionally, every data source is represented in the virtual world, where it can also store information (see Figure 1).

Besides spatial and administrative relationships, Figure 1 also shows an example of the interaction between data sources and agents playing diverse roles. The procedure is as follows:

1. The inquirer requests some information from the data source.
2. The data source, according to privacy rights, returns the answer to the inquirer. The answer may contain the whole data or it can be filtered. In the latter, the inquirer will only receive a partial view of the data.
3. When the answer is delivered, other agents or users may receive it. This may happen with or without an explicit indication of the inquirer. For instance, an answer may be broadcast to unsolicited receivers if a speaker is used as the output device.
4. Once a user obtains data from a source, he may freely distribute it to other users.

The privacy management approach presented in this paper deals with the first and second phase. The third and fourth phases are beyond the scope of this work.

### 3.2. Taxonomy of Personal Information

Information is probably the most important element of privacy. In fact, privacy can be summarized as protecting information loosely enough to permit interaction, but sufficiently tight to preserve confidentiality. According to the nature of the user's personal data, we classify information into two categories: long-term information and short-term information. The former comprises information with a low change rate, e.g., telephone number, address, social security number. The latter, on the other hand, contains information of a changing nature, e.g., identity, location, activity. These two types of information have their own strengths and weakness. Thus, since long-term information is probably valid in the future, a single unwanted access will endanger the information in the long term. Consequently we can categorize this information as especially sensible in a time line. On the contrary, short-term information may change with time, for which it could seem less sensible, on the other hand, to precisely know if this kind of information really describes a person's way of living, e.g., where you are, what you are doing what and with whom. Therefore, even though not especially sensible in a time line, this kind of information is more directly related to what we understand by privacy than the previous one.

Long-term information is insensible to context changes, meaning that once the value is revealed to an unwanted receiver, the data are compromised forever. In this case, the main concern must be who has rights to access the information, while when, where or how it is accessed is not relevant. Accordingly, the privacy mechanism associated with long-term personal data is based on a restrictive approach, in which the user must specify which individuals can access each piece of long-term information.

Contrary to long-term information, we consider only the three context variables types of Dey [36] for short-term information: identity, location and activity. Even though identity can be considered, as it is in fact, as long-term information, it must be also kept as short-term, due to the indexing function it bears. Thus, without identity, neither location nor activity have any sense.

Short-term information is classified according to two discrete axes: privacy level and personal data. The accuracy of the information varies with the privacy level. This accuracy is characterized by different degrees of granularity of each variable. Although different scales and values can be considered, our three-level approach is motivated by the simplicity of use, in other words remaining simple enough to be comprehensible by non-technical user. The three privacy levels are:

– Restricted: At this level, a non-disclosure of information is achieved. Thereby, user's information is not distributed to the community.
– Diffuse: Personal data can be retrieved with some level of distortion. Thus, for each variable, an intermediate value is established hinting at the real one without revealing it.
– Open: The information is revealed without any modification. Hence, the community receives the information as accurately as possible. Besides privacy configuration, the accuracy of the data will depend on the sensors' quality and deployed infrastructure.

The main challenge relies on how to assign these privacy levels to short-term information. One possible approach would be to apply the same strategy as for long-term information. However, due to the constantly changing nature of context, an approach that requires full control over privacy will force the definition of particular privacy settings for each situation, meaning that users must anticipate every possible situation or review their privacy preferences every time the situation changes. While the former is non-feasible, the latter can be achieved by following an incremental approach, with the system asking for new privacy settings every time a new situation occurs and then remembering it. In this way, the system acquires, step-by-step, privacy settings close to the ideal. A real example can be found in firewall applications. On the other hand, the main problem with this kind of solution is that users might find it annoying, as truly is case of firewalls, to have repeated interruptions of the system, even more if we consider that notifications that might be sent through ambient interfaces, such as speakers. A completely opposite approach would be to consider totally automatic privacy control as an option. This implies that the system automatically infers the privacy configuration for each situation for behavior of the user, who stays aside. However, we believe that it will be hard for end-users to feel comfortable in delegating control over their privacy to an automatic system, being unable to explain its behavior [37]. Although automatic decision control techniques can be very competent, user direct control is particularly relevant when the variables to be controlled are personal data.

Summarizing, and following Maes' [38] guide on trust and competence as the main issues in developing software applications, we would like to emphasize the importance of choosing a comprehensible and useful solution, where comprehensible means that the user has enough knowledge of the how, when and why the system reacts to trust it; and a useful means that the system is sufficiently easy to use and powerful enough to achieve a high degree of competence over the problem.

### 3.3. “Fair Trade Metaphor”

Our proposal relies on the following assumption:

“Users will accept harm to their privacy if, on return, they receive valuable services and they are able to track the flows of their disclosed information.”

Our information management protocol is among those referred to as “fair trade politics”; in other words, a user can see another user's context variable (identity, location, activity, time), with the degree of privacy established by the owner, if he harms his privacy equally. This model comprises only what we called short-term information for which opening or closing the long-term information to some, none or all others is up to the information's owner through manually configuration.

For example, following the previous approach, if a user defines his privacy level as diffuse, his personal data will be shown after being filtered, but he would only be able to retrieve filtered information from others in the best case, even from those with an open privacy level. At the end, the amount of shared information grows information needs, making context-aware services more useful and valuable.

Therefore, the information retrieved by the inquirer is filtered depending on the data source privacy level. This level is automatically updated according to the inquirer's and user's privacy levels following Equation (1).

(1) $${\text{PrivacyLevel}}_{\text{data}}=\mathrm{Min}\left({\text{PrivacyLevel}}_{\left(\text{inquirer}\right)}{\text{PrivacyLevel}}_{\left(\text{user}\right)}\right)$$

The model stresses “fair trade”. Thus, a user will set his privacy according to the precision level in which he wants to see others' information, assuming that others will be able to see his/her own equally. For instance, a user setting his privacy level to diffuse will only have access to others' information with the same degree of granularity as much, even if they have declared it as “open”. In Table 1, we provide several examples for how different variables are filtered depending on the resulting privacy level.

Different mechanisms are used to support our privacy control system. An optimistic access control allows free access to information, with strict registration of whom, what and when accedes to it. This registration acts as a dissuasive means to prevent abuses within the system.

Templates, combined with default configurations, help users in controlling their privacy and modifying it when necessary. The goal is to promote user's interest in protecting his/her long-term information in a practical and simple way. Thus, if a user wants to show his/her telephone number, he/she will explicitly specify, through a template, to which user or group of users it must be shown. Contrary to short-term information, templates for long-term information use a restrictive configuration.

Optimistic access control provides a logging service to keep users informed about who accesses their information and to what degree. This mechanism provide a way to detect inappropriate, suspicious or abusive conducts (for instance, to detect a user consulting activity and the location of others excessively in the course of the day).

The punitive measures can consist of applying a restrictive privacy to that specific user, expelling him/her from the users' group of confidence and, as a consequence, limit the quality of services he/she may receive. Additionally, a voting system could be applied in which those conforming to the community maintain a list of unpleasant users to which deny services in the environment.

The value of personal information depends on each person. Thus, independently of the information nature, a manual procedure must be provided to the user for modifying his/her privacy.

## 4. Implementation

There are multiple privacy definitions, according to the period and type of problem to which it is applied; however, we can say that privacy always is a matter of values and interests. A definition of privacy that is well suited to our research objectives is described by Alan Westin [39].

“The claim of individuals, groups, or institutions to determine for themselves when, how, and to what extent information about them is communicated.”

The importance of the above definition, is in the following assertions:

– Privacy as a right of the information owner: it is the right of individuals to determine for themselves for who (one user, one group, all users), where (disclosure place), how (disclosure medium) and what (information use) private information is disclosed.
– Privacy as a information property: a feature set by the owner of the information, opening the possibility that other users can access it.

According to the first assertion, we can identify five key elements for our model: owner, receiver, context, communication medium and use.

– Owner: He decides how the privacy of the information is managed. The owner may be an individual or a group (there are several ways to manage your privacy: unanimously, majority and the most restrictive option).
– Receiver: He has the right to see the information. The receiver can be a person or a software component. This requires an authentication mechanism to ensure that the receiver is the true destination of the information.
– Context: Another important factor is the context of the source entity, like a destiny entity. The privacy settings vary depending on where the information is disclosed, where the owner of the information is (public or private place), the number and people at that time that are the receivers of the information.
– Communication medium: The communication medium used to disseminate information becomes important for the owner of the information; the privacy restrictions are different depending on the medium of communication used.
– Use: The receiver of information can misuse it (store, reproduce or disclose without the owner's permission).

According to privacy as an information property, privacy is an attribute of information that can be manipulated by its owner to restrict disclosure. In our model, the information's owner has the option of configuring their information as private or public, depending on the receiver and context assessment.

To implement the privacy statements on an active environment, we must have some means of showing existing entities and the context.

### 4.1. Information Modeling

In this section, we define the terminology used in our active environment. The context representation includes some ideas expressed by Dey [36] to represent people, places and resources, through concept definitions (or classes) used as templates for the creation of instances, also called entities.

Each entity belongs to a concept and is represented by a name and a set of properties. Each property has a name and a value; it can be literal or some other entity. In this case, a relationship between the first entity and the second is set. Relations are unidirectional, although for each relationship, the inverse can be defined.

Finally, a set of parameters can be defined and associated with a concept, entity, property or relationship.

### 4.2. Context Layer

A context layer acts as an interface between different computing devices, towards the integration of an active environment [40]. A real-world centralized model is the best means of achieving the interaction between devices.

The context layer provides, first, a repository where entities are stored and, second, an interface with a function to make an abstraction of the protocol's communication details with physical devices.

The context layer implementation can be seen as a global data structure, called a blackboard [41]. This blackboard stores an abstract model of the world where the most important information regarding the environment (including users) is stored. Therefore, each device, user and resource of the active environment will be represented by some entity on the blackboard. Additionally, it contains a representation of the existing information flow between physical devices (microphones, speakers, cameras, displays, *etc.*).

Blackboard information is used by heterogeneous devices to understand the context and to adapt to it. Each blackboard is a server that can be accessed by the TCP/IP protocol. HTTP has been chosen as the transport protocol for its simplicity and wide coverage. For information exchange between applications and the blackboard server, the XML language is used.

The blackboard provides the following basic operations: Add (dynamically add entities to the blackboard), Remove (reciprocal to Add), Get (ask for blackboard values), Set (change one value on the blackboard and report this change to the context, in other words, make a change in the physical world), AddRelation (capture contextual information through the relationship between entities), RemoveRelation (reciprocal to AddRelation), Subscribe (an application is notified from blackboard changes) and Unsubscribe (an application is no longer notified of context changes).

### 4.3. Middleware Implementation

**Figure 2:**
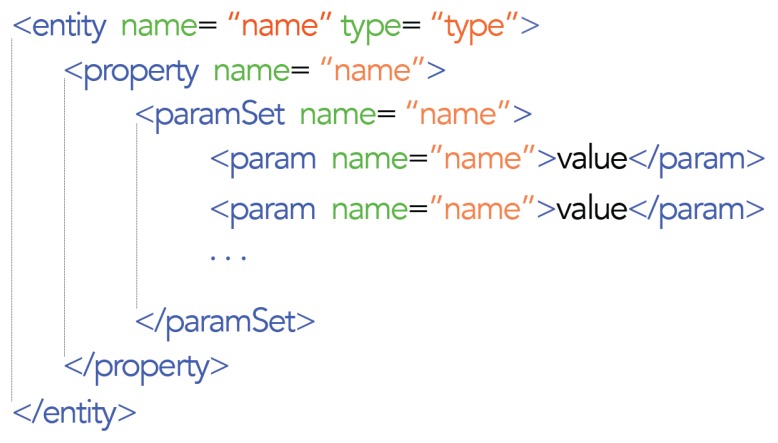
XML entity's representation.

As mentioned in previous sections, the entities that make up the blackboard are represented in XML. In Figure 2, you can see the entity basic parts, formed by properties and relationships, and parameter sets, ParamSet, to complement the entity's definition.

The implementation of our proposed privacy is based on adding a set of parameters called privacy [42]. This set delimits the privacy of the entire entity or one property. The different parameters that are part of privacy set are described. A privacy manager was implemented, who is assisted by four parameters: type, receivers, environment and use.

The first one (type), delimits the information's scope. The owner may classify information into three different types:

– Private: a private entity, property or relationship that will only be accessed for his/she owner; therefore, no one else can access it.
– Public: a public entity, property or relationship that will be accessed by other entities or relationships, able to consult or modify it.
– Protected: a protected entity, property or relationship that will have an entities list (prepared by the owner) with allowed access.

It is necessary to ensure that the information is delivered to the correct receiver. Therefore, a receiver parameter was integrated; here, the entity's owner defines the entity identifiers that are allowed access. The, identifiers that can go inside the receiver parameter can correspond to an individual entity (a person or software component) as a group. When an entity wants to access the resources of another, it is checked if its identifier is within the receiver parameter or if it is in one of the groups included in this parameter.

Another element is the location owner information. A third parameter called environment was integrated, in which the entity's owner defines places subject to the privacy's parameters being applied.

A third parameter called environment was integrated, in which the entity's owner must define all of the places subject to the application of a set of privacy parameters. Therefore, when somebody wants to access some information, first the owner's location must be verified, and depending on the location, a set of privacy parameters should be applicable for this environment.

Finally, a fourth parameter called use was added, and its function is to restrict the use of information once the receiver has already acceded to it.

The use of the information was classified into three different types:

– Recording: permission granted (by the entity's owner) to the receiver for storing a copy of the information.
– Broadcasting: permission granted (by the entity's owner) to the receiver for disseminating the information to other entities.
– Viewing: permission granted (by the entity's owner) to the receiver to view the information.

Thus, the receiver can be restricted by the use that can be made of the information, once permitted.

The information represented on the blackboard (context) has two attributes: the first one read-only and the second to be able to modify (write). Previously it was mentioned that a list of entities and groups can access the information through receiver parameter. Therefore, each entity or group identifier is concatenated to permit read and/or write information(+r, +w, +rw).

**Figure 3:**
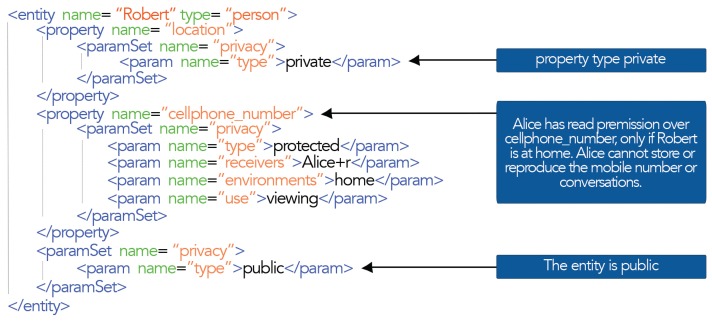
An entity with privacy settings.

An example of how to add the privacy model to the blackboard entities can be seen in Figure 3.

As shown in Figure 3, the entity defines a global privacy; therefore, its scope extends to their properties and relationships defined within it. In the example, the entity was configured as public, which means that any entity could access the properties and relations of the entity. However, the location property is set to private, which restricts access to that property for all receivers. Moreover, *cellphone_number* is set as a protected property. This implies that it can access only if the restrictions imposed by receiver parameters, environments and use are met.

The privacy of a relationship can also be set. If the relationship is private, it will not be shown to the receiver when accessing the entity. However, if the relationship is public or protected, the receiver will know the existence of the relationship. Therefore, in a relationship, a destiny entity may have a different privacy than the source entity; therefore, the receiver can access the information's source entity, but not the information of the destiny entity.

## 5. Evaluation

To validate our hypothesis, we developed a context-aware privacy-sensitive instant messenger with which to evaluate how feasible the “fair trade” metaphor is as a privacy control mechanism. The prototype runs on top of the infrastructure deployed in the Universidad Autónoma de Madrid [40] and Instituto Tecnológico Superior Zacatecas Norte; a working version is being used daily by the research staff of our group. We are working on making an evaluation on a broad variety of end-users, in order to determine whether the number of different privacy levels has to increase or not and if the user's black lists should be public.

The goal of our study is to evaluate the possibility of encouraging users to share their private information, promoting the use of contextual services by the community. The instant messenger was developed under the “fair trade” metaphor and was used as a means of communication between habitants of two real active environments.

The proposed methodology is as follows:

– A previous interview of the candidates and pre-questionnaires
– Installation of the instant messaging client
– Use on a daily basis (two weeks approximately)
– Second interview (feedback effects) and post-questionnaires
– Recoveries of the transaction log file for review.

The interview consists of three sections. The first one contains some questions, which attempt to determine the perception of privacy by the user, classifying different kinds of information according to his/her importance. The second one determines the preferred privacy control mechanisms by the user, and finally, the candidate makes an assessment of the mechanisms available for the system in order to be alerted when someone accesses his/her private information.

A call for participation was launched for computer science students of Instituto Tecnológico Superior Zacatecas Norte, related to the experiment called “privacy perception in active environments”. Applicants must have a computer, Internet connection, availability for installation and use an instant messaging application. Twenty one students were selected, all frequent users (daily use) of IM applications (except two, who reported using it at least a couple of times a week), with ages ranging from 19 to 24 years old; 14 were men and seven women (see Table 2).

The initial interview evaluated the perception of user privacy. It was important to get the user's opinion regarding:

– The factors involved in establishing the level of privacy and the order of importance in a given situation.
– Information considered sensitive (the importance of being disclosed to third parties) and utility (advantage of knowing certain information from a third party).
– Privacy control (control mechanisms, receivers, granularity, optimistic/pessimistic policy).
– Feedback (system alert notifying you that someone has accessed your private information).

The second interview was applied as a means of feedback in order to evaluate the ease of use, learning and satisfaction of the IM application, but mainly to know the perception of the usefulness of the proposed privacy management.

**Figure 4:**
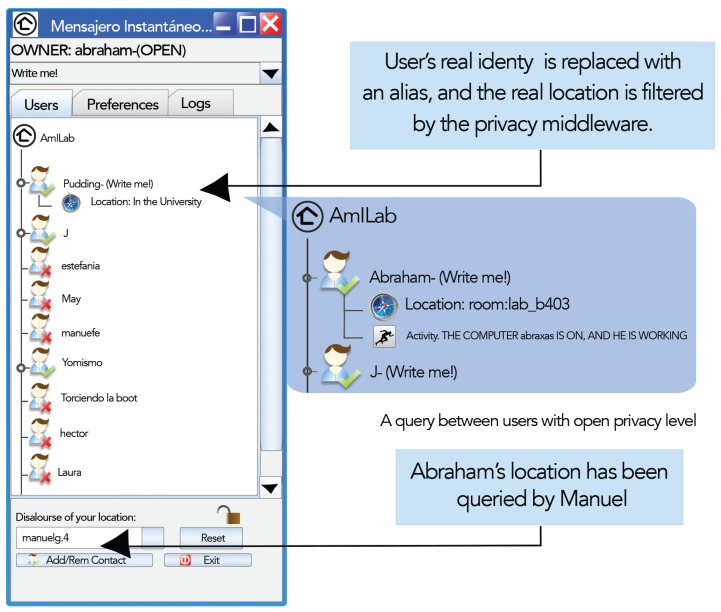
Instant messenger.

Since sensing technologies might not be available in every place, the IM client allows configuring an alternative version of the location and activity values manually. Nevertheless, information obtained directly from sensors will always be preferred to information stated manually, in order to avoid conscious chatting from malicious users. In AmiChat, short-term information is defined in terms of location and activity, and the three privacy levels defined in Section 3 (see Figure 4) are incorporated to the IM:

– Restricted: Privacy has the highest level of closeness. Personal information remains secret; as a consequence, the user loses IM quality of service. When the user connects to the IM, his/her identity will be shown as “anonymous”, as they will be all of their buddies' identities, as well, regardless of their privacy configuration. Despite this lack of context information (identity, activity and location), the IM service remains active, so the user can send and receive messages even though he does not know to whom or from whom.
– Diffuse: At this privacy level, information is retrieved with certain granularity. User's real identity is replaced with an alias. As a consequence, the user will only view the alias of his/her buddies with diffuse or open privacy levels. The activity to be shown is selected, as in many commercial IMs, from a list with three different options: “Write me !!”, “Away” and “Don't disturb”. Regarding location, the result value is obtained from environment sensors after being filtered by the privacy middleware. Location information is gathered from two different kinds of sensors. On the one hand, working spaces, such as offices and laboratories, are outfitted with RFID readers. Users coming in and going out of the environment should pass their RFID cards. The IM server is notified of events on changes in locations. Additionally, a spatial model provides a hierarchy of environments, including relationships between them, such as inclusion or adjacency. On the other hand, IM monitors the user's personal computer status by means of a resident program that informs if the user is connected or is away. The previous spatial model also represents an inclusion relationship between resources and environments. Thus, if a user is using her PC, the IM can infer where she is.
– Open: Personal information is completely open; hence, the user is able to see others' information with the highest possible resolution: the one defined by the information owner. The identity shown is the real one, and the location and activity values are directly inferred from sensors (e.g., “At the computer, but not working” or “At Lab Room b403”). Thus, personal information is traded for quality of service.

### 5.1. Results

Data for this study were collected using a five-point Likert-type scale. The conclusions are obtained from the mode (the most frequent response) that can be seen as the highest percentage for each tuple.

Regarding the perceived usefulness of the “fair trade” metaphor for privacy control in the instant messaging application, the possible answers are (see Table 3): 1, not useful; 2, sometimes useful; 3, useful; 4, often useful; 5, very useful.

The possible responses in terms of ease of use, are: 1, is very hard; 2, sometimes hard; 3, sometimes easy; 4, often easy; 5, very easy (see Table 4).

For learnability of the metaphor in the IM application, users responded (see Table 5): 1, strongly disagree; 2, disagree; 3, neutral; 4, agree; 5, strongly agree.

The satisfaction degree was derived from the following questions (see Table 6), with the following possible answers: 1, does not satisfy me; 2, sometimes satisfied; 3, neutral; 4, satisfied; 5, strongly satisfied.

The most important part of the second interview is in the perception of privacy control (see Table 7), where the following results were obtained from these answers: 1, strongly disagree; 2, disagree; 3, neutral; 4, agree; 5, strongly agree.

The factors according to user responses that are relevant to determining their level of privacy in a given situation are (beginning from the most important to the lest): (1) where the information is stored; (2) the situation; (3) content, *i.e.*, the meaning of the information; (4) receiver; (5) the role that played in the situation: parent, child, boss, employee, teacher; (6) retention, *i.e.*, how long it takes until the information is removed; (7) information use by the receptor; (8) who is with the receiver; (9) who is with the owner of the information; (10) the location of the information's owner; (11) the location of the information's receiver; (12) the hour of the day in which information is consulted.

Regarding the sensitivity of the information (importance to be disclosed to a third party), the results (beginning from the most important to the least) are: phone number, identity of people around me, location (where I am), name and last name, company where you work, address, main email account, occupation, date of birth and what I am doing. The privacy preferences also depend on cultural and social aspects. Some states in México have a high incidence of crime, such as extortion and kidnapping, which can be seen in the value of certain information, such as the phone number and identity.

Concerning the control mechanisms for managing privacy, three proposals were provided: (1) manually (privacy would be set *a priori* for each situation); (2) trained (for each access of information in a different situation, the privacy level would be asked, and it will be stored for future use); and (3) automatically (the system decides the level based on profiles or behavior patterns). The result was that for long-term personal information, such as identity, address and phone number, there is a trend towards manual administration. On the other hand, for the location information (where I am) or what I am doing, which is considered short-term personal information, in the second interview, the user motivation was changed from manual to a trained or automatic configuration.

>From the results, it can be appreciated that the user prefers making changes, depending on the situation, at the level of detail of his/her short-term information, as in the case of location, the activities the user is performing and occupation.

## 6. Conclusions

The findings provide support to the theoretical framework of this study and indicate that, as a result of the interviews, we can conclude that users find the classification of information (short-/long-term information) useful. Regarding short-term information, the user prefers to maintain strict control (restrictive configuration) through manual administration, over the disclosure of his information.

One goal of this study was to classify information according to its importance. According to our results, the long-term information value and its classification is directly dependent on cultural and social aspects.

With regard to the short-term information, the study shows that variables like identity, location and activity are appropriate to publicize the dynamic context of a user.

Given the variety of situations that may arise in the context of an active environment, the users prefer a short-term information management through trained or automatic control mechanisms (based on templates, combined with default configurations), controlling the exposure information degree (granularity), even to a receivers' group.

After using the application and the second interview, users were willing to use an optimistic policy for the treatment of short-term information if, on return, they receive valuable services and they are able to track the flows of their disclosed information.

Interesting results were obtained from the development of applications based on fair trade mechanisms, where users exchange their personal data in a symmetric way and users are both consumers and producers of personal information.

As future work, it would be necessary to implement punitive measures to punish optimistic access control abusers, such as a blacklist. The development of new applications under the “fair trade” metaphor involves sharing the blacklist for denying services to abusers until the community revokes the punishment.

Even when the goal of our study is to evaluate the possibility of encouraging users to share their private information promoting the use of contextual services by the community, we have presented a slight evaluation of the IM application regarding utility and ease of use, with respect to the fair trade metaphor. A more formal study based on a technology acceptance model is needed for a complete assessment.

Another pending issue is investigating the interaction process between the context layer (blackboard), the fair trade metaphor and a directory service, as a mechanism for managing authentication, authorization and accounting.

## Figures and Tables

**Table 1:** Context variables are filtered using different granularity depending on the privacy level.

**Privacy Levels**	**Identity**	**Location**	**Activity**
Restricted	Anonymous	Not available	Not available
Diffuse	Alias	Building level	High level activity
Open	Real	Room level/GPS data	Raw data

**Table 2:** Demographic attributes of the respondents.

	**Frequency**	**Percent (%)**	**Cumulative**
Gender			

Male	7	33.33	33.33
Female	14	66.67	100.00

Age			

19	6	28.57	28.57
20	6	28.57	57.14
21	4	19.05	76.19
22	1	4.76	80.95
23	3	14.29	95.24
24	1	4.76	100.00

Frequency of use of an IM			
Daily	19	90.48	90.48
At least a couple of times a week	2	9.52	100.00

**Table 3:** Perceived usefulness of the “fair trade” metaphor for privacy control in the instant messaging application.

	**1**	**2**	**3**	**4**	**5**
(a) Is useful	0.00%	0.00%	9.52%	33.33%	57.14%
(b) It saves my time when I use it	0.00%	0.00%	14.29%	38.10%	47.62%
(c) Meets my needs	0.00%	4.76%	38.10%	42.86%	14.29%
(d) It does everything you could hope it would do	0.00%	4.76%	38.10%	42.86%	14.29%
(e) It provides control over the activities of my life	0.00%	4.76%	33.33%	33.33%	28.57%
(f) The environment that I want to accomplish is done in the simplest way possible	0.00%	9.52%	14.29%	23.81%	52.38%

**Table 4:** Responses in terms of ease of use.

	**1**	**2**	**3**	**4**	**5**
(a) It is easy to use	0.00%	0.00%	14.29%	19.05%	66.67%
(b) It is simple to use	0.00%	0.00%	0.00%	28.57%	71.43%
(c) Performing a task requires fewer steps	0.00%	0.00%	4.76%	52.38%	42.86%
(d) It is flexible	0.00%	4.76%	14.29%	19.05%	61.90%
(e) Using the application does not require much effort	0.00%	0.00%	9.52%	19.05%	71.43%
(f) I can use the application without reading a manual	0.00%	0.00%	14.29%	57.14%	28.57%
(g) I cannot find contradictions in its use	0.00%	0.00%	14.29%	57.14%	28.57%
(h) Regular and irregular users would like to use it	0.00%	4.76%	19.05%	23.81%	52.38%
(i) I can resolve faults quickly and easily	0.00%	0.00%	38.10%	33.33%	28.57%
(j) I can use it successfully anytime	0.00%	4.76%	14.29%	23.81%	57.14%

**Table 5:** Results in terms of learnability of the application.

	**1**	**2**	**3**	**4**	**5**
(a) I learned to use it quickly	0.00%	0.00%	0.00%	14.29%	85.71%
(b) I easily remember how to use it	0.00%	0.00%	4.76%	14.29%	80.95%
(c) It is easy to learn to use	0.00%	0.00%	9.52%	9.52%	80.95%
(d) I quickly became skilled in its use	0.00%	0.00%	4.76%	14.29%	80.95%

**Table 6:** Results in terms of satisfaction degree.

	**1**	**2**	**3**	**4**	**5**
(a) I am satisfied with the application	0.00%	0.00%	19.05%	28.57%	52.38%
(b) I will recommend it to a friend	0.00%	0.00%	19.05%	23.81%	57.14%
(c) Using it is fun	0.00%	0.00%	19.05%	33.33%	47.62%
(d) It works as I want it to	0.00%	4.76%	28.57%	28.57%	38.10%
(e) It is wonderful	0.00%	9.52%	28.57%	23.81%	38.10%
(f) I think it should be mine	4.76%	4.76%	19.05%	23.81%	47.62%
(g) It was a pleasant experience to use	0.00%	0.00%	14.29%	28.57%	57.14%

**Table 7:** Perception of privacy control.

	**1**	**2**	**3**	**4**	**5**
(a) The difference between short-term and long-term personal information was clear	0.00%	0.00%	4.76%	33.33%	61.90%
(b) The privacy mechanism allowed my identity, location and activity to remain at a safe level of disclosure	0.00%	0.00%	0.00%	28.57%	71.43%
(c) The application goes according to the active environment services	0.00%	0.00%	23.81%	28.57%	47.62%
(d) Short-term privacy preferences were reliable despite who was with me when using the application	0.00%	0.00%	14.29%	38.10%	47.62%
(e) Privacy preferences change according to the time of day when a user uses the application	0.00%	19.05%	19.05%	28.57%	33.33%

(f) You had control of your location at any time	0.00%	0.00%	9.52%	23.81%	66.67%
	0.00%	0.00%	9.52%	9.52%	80.95%

(g) You had control of your telephone number at any time	0.00%	0.00%	14.29%	23.81%	61.90%
(h) You had control of your activity (what you were doing): working, resting, busy, *etc.*	0.00%	0.00%	4.76%	33.33%	61.90%
(i) You had control of your identity at any time	0.00%	0.00%	9.52%	23.81%	66.67%
